# Medical management of glaucoma: Principles and practice

**DOI:** 10.4103/0301-4738.73691

**Published:** 2011-01

**Authors:** Kuldev Singh, Anurag Shrivastava

**Affiliations:** Glaucoma Service, Stanford University School of Medicine, 900 Blake Wilbur Drive, Palo Alto, CA 94305, USA; 1 Montefiore Medical Center, Albert Einstein College of Medicine, Bronx, NY 10467, USA

**Keywords:** Glaucoma, maximal medical therapy, medical management, target intraocular pressure

## Abstract

Glaucoma care is more an art than science. The introduction of several new classes of glaucoma medications and the completion of many large randomized clinical trials have not changed this fact. While we now have better choices when initiating glaucoma therapy relative to our predecessors, the principles of glaucoma therapy have not changed much during this period. Debates continue regarding the utility of concepts such as “the monocular therapeutic trial,” “target intraocular pressure (IOP),” and “maximal medical therapy.” Our tools for detecting and following glaucomatous disease have improved but are not precise enough for us to prospectively predict which patients will do better or worse than others. Much attention has been given to disease stage, rate of progression, and compliance with medications but regular patient follow-up, an area that has received little attention, may be among the most important predictors of patient outcomes.

A Viennese ophthalmologist once commented: “there are two types of glaucoma patients, those who do well no matter what you do for them and those who do poorly no matter what you do for them.” While this may be an oversimplified summation of glaucoma care, it is one perspective which can be useful in reminding us of the importance of disease staging. When the initial diagnosis of glaucoma is made, the practitioner will do well to assess the likelihood of the patients noticing vision loss from the disease over the course of their lifetime. In developed countries, glaucoma is both undertreated and overtreated. It is undertreated in many who present at advanced stages of disease at a young age in whom very low intraocular pressures (IOPs) should be obtained as soon as possible. Medical glaucoma therapy for such patients is commonly unsuccessful and early surgical options should be considered. In contrast, patients presenting with ocular hypertension or early glaucomatous disease, particularly those who are elderly, should obviously be treated less aggressively. The setting of low-IOP goals in such patients can be hazardous, with the potential to do more harm than good. Both medical therapy and, in some instances, observation without therapy is the better approach in this latter group of patients who are likely to do well “no matter what you do for them.”

The developing world paradigm is, of course, made more complicated by the fact that not all treatment options are available in many environments. The cost of medications and obstacles to distribution make medical therapy for glaucoma impractical and, sometimes, impossible in some developing world settings. Given the lack of availability of medications and laser trabeculoplasty as well as the significant risk involved with surgical glaucoma therapies, there exist glaucoma patients with mild disease and low risk of vision loss in whom the best option is to simply follow the patient carefully without therapy to observe rates of progression in certain, but not all, developing world situations. The decision to proceed with trabeculectomy or drainage device implantation is a big step as initial therapy, the risks of which should not be taken lightly. Thus while glaucoma is primarily a surgical disease in many developing world countries, not all glaucoma patients should undergo glaucoma filtration surgery in such settings.

In this paper, we provide an overview of the principles and practice of medical management of glaucoma patients. In doing so, we make the assumption that practitioners have access to all contemporary classes of glaucoma medications and that the cost of obtaining such medications does not create such patient hardship that the practitioner is substantially hampered in his or her ability to care for those with the disease. We acknowledge, however, that this assumption is unrealistic. Many of the principles of management are based upon the results of a modified RAND-like methodology which was used to develop consensus around the topic of glaucoma management.[1,2]

## Initiation of Medical Therapy

The Collaborative Initial Glaucoma Treatment Study (CIGTS) showed that there was no difference between initial medical versus surgical therapy in visual preservation but that subjects preferred medical therapy primarily because the side effects associated with initial surgical therapy are more troublesome than those found with medical therapy.[3] Overall, initial medical therapy remains the treatment of choice for most patients with open angle glaucoma.

The prostaglandin analogs are the preferred first agents for glaucoma therapy for a variety of reasons. These agents lower IOP extremely well when dosed once a day and this effect has been shown to be long lasting without significant tachyphylaxis.[4] The diurnal and nocturnal IOP lowering of prostaglandin analogs has been found to be superior to all other topical classes of glaucoma medications. In particular, prostaglandins lower IOP to a greater extent than timolol in the nocturnal period as demonstrated in several 24-h studies.[5,6] This is particularly important given that IOP is highest in the nocturnal period for most patients with glaucoma or ocular hypertension when measured in habitual body positions: supine at night and sitting during the day.[7] Another positive aspect of the prostaglandins is the fact that this class lowers IOP beyond 24 h thus compensating, to some extent, for patients missing occasional doses of medication.[8,9] While beta blockers are associated with a host of systemic side effects, the prostaglandin analogs are extremely well tolerated systemically with no serious adverse consequences in most patients. The exception is during pregnancy, when these agents are contraindicated due to the potential risk of miscarriage. The degree of risk during pregnancy has not been well elucidated.

While extremely safe systemically, the prostaglandin analogs do have several associated ocular side effects including darkening of iris color, lash growth, periocular skin pigmentation, and hyperemia.[10] The hyperemia is seen with initial instillation and does not appear to be a classic allergic reaction that is seen so commonly with other classes of drugs. True allergy to the prostaglandin analogs is rare. Other potential side effects include worsening of intraocular inflammation, cystoid macular edema, and reactivation of corneal herpes virus infections.[4,11] The evidence in support of these infrequent side effects is weak.

The primary reason for choosing a medication other than a prostaglandin analog for initial medical therapy is the potential for ocular side effects in some patients. In those of European ancestry, eye color change from blue to brown may be an unacceptable risk, particularly in patients receiving monocular IOP lowering therapy. This is obviously an insignificant issue in brown-eyed individuals. Patients with blue-brown or hazel eyes are at greatest risk of iris color change. The periocular skin pigmentation seen with prostaglandins can be concerning both light- and dark-skinned individuals.

In circumstances where prostaglandins are contraindicated due to concerns about ocular side effects or patient unwillingness to accept the risks of such side effects, the nonselective beta blockers dosed once or twice a day are acceptable alternatives. This class of drugs is unmatched with regard to ocular tolerability when it comes to glaucoma therapy. Most cases of allergy to beta blockers are due to preservative sensitivity rather than intolerance to the active component drug. The downside of beta blockers relative to the prostaglandin analogs relates to the quality of IOP lowering of the former group and the significant systemic side effects seen with this class of drugs. Beta blockers have minimal, if any, effect on IOP lowering in the nocturnal period[12] and the IOP lowering effect of these drugs, regardless of whether they are dosed once or twice a day, is not as long lasting as that found with the prostaglandins. Thus noncompliance with medications is likely to have a greater impact on long-term IOP lowering when beta blockers rather than prostaglandin analogs are used as initial glaucoma therapy. The systemic side effects of beta blockers are legendary. Bradycardia and bronchial disease are two absolute contraindications, regardless of whether one considers selective or nonselective beta blockers. While congestive heart failure was previously considered a contraindication for beta blocker use, oral beta blockers are now included in the medical regimen commonly used to treat this condition.

The role of other contemporary classes of glaucoma medications, including topical carbonic anhydrase inhibitors, alpha adrenergic agonists, and parasympathomimetics is limited for first-line use due to the necessity of using these drugs more than twice a day and because the quality of IOP lowering, even when dosed three or four times a day, is not as desirable as that seen with the prostaglandin analogs. In rare circumstances when both prostaglandins and beta blockers are contraindicated, these other agents may be used as first-line therapy.

The monocular therapeutic trial has been the standard recommended practice when initiating glaucoma therapy for many years. The adherence to this recommendation has varied among practitioners. In recent years, the relevance of the monocular therapeutic trial has been challenged by the notion that IOP does not vary symmetrically in the two eyes.[13] IOP fluctuates over the diurnal and nocturnal period and one of the greatest challenges in assessing the response to medical therapy is the lack of a good baseline IOP prior to initiating treatment. When one does not establish a baseline that is reflective of the patient’s true IOP, the assessment of response to medical therapy becomes very difficult. Thus getting several IOP measurements on different days and times may be very useful prior to initiating medical therapy, particularly, if this can be done practically without putting the patient at risk for vision loss. The monocular trial becomes less useful in circumstances when a good baseline IOP has been established. It is also less useful when initiating therapy with agents that are associated with a high likelihood of response such as the prostaglandin analogs and beta blockers. Practically speaking, it is likely that most practitioners do not begin therapy with a monocular trial, unless, of course, therapy is only indicated in one eye. The monocular trial may, in fact, be more useful in discontinuing rather than initiating therapy.

## Setting a Goal

The glaucoma world has been dominated by the dogma of target IOP for several decades.[14–19] The concept was, unfortunately, reinforced by a post hoc analysis from the Advanced Glaucoma Intervention Study which led some to propose that lowering IOP to below 18 mmHg on all visits or an average IOP of 12.3 mmHg would halt glaucoma progression.[20] This concept has further propagated a binary, all or nothing, approach to glaucoma care which simplifies the practitioner’s life but does not necessarily improve patient care. The two components of this binary approach relate to reaching a target IOP goal and structural/functional progression of glaucomatous disease. It is assumed that when the IOP is above “target,” the disease will be progressive, and furthermore when it is below target, glaucoma progression will halt. Based upon this dogma, if one has achieved the target goal and the disease continues to progress, then one must set a new lower target.

One reason why this approach has been so popular is that it allows the practitioner to have an abbreviated dialog with the patient. It takes just a few minutes to tell the patient that the IOP is below target and that no further treatment is indicated or that more treatment is needed because the IOP is above target and/or the visual field/optic nerve has shown progressive glaucomatous change. We know, of course, that glaucoma progression is not an all-or-nothing phenomenon. Every patient is losing retinal ganglion cells on a daily basis and thus the disease is always progressing. It is the rate of progression that is important and, undoubtedly, this rate is generally lower when IOP is lower in most circumstances. Unfortunately, however, it is impossible to prospectively know the IOP rate of a progression relationship in an individual patient and this relationship may not always be clear retrospectively as well. Thus the target IOP is at best an educated guess and more likely an arbitrary choice for a particular patient. While this may appear to some as an improvement from choosing the same target, 21 mmHg, in all patients as was the case in the past, individualized target IOP determination still leaves much to be desired.

**Figure 1:**
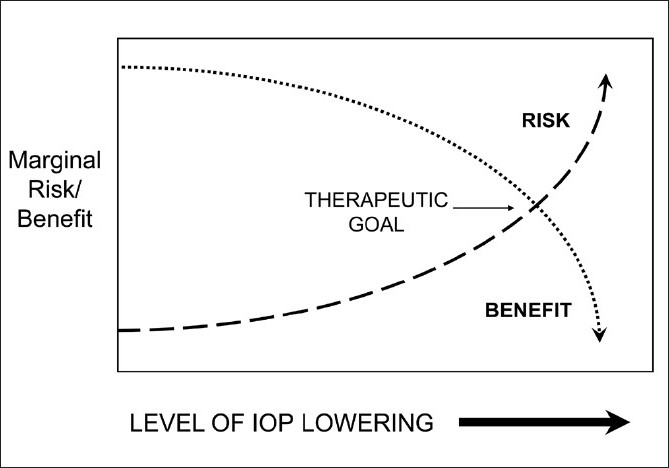
The therapeutic goal of glaucoma management: balancing risks and benefits

The target IOP concept has several other limitations.[21,22] Glaucoma care involves balancing risks and benefits of therapy and the definition of target IOP[23] does not mention anything about risks or side effects. While experienced practitioners may modify the target IOP based upon the potential side effects of reaching the goal, this flexible approach is not part of the definition of the concept, and practitioners who rigidly adhere to a target level or range may potentially do more harm than good in many circumstances. For example, an elderly low-risk patient with ocular hypertension or mild disease who is, for whatever reason, unable to reach a target IOP goal with medical therapy and/or laser trabeculoplasty, should generally not undergo glaucoma filtration surgery to reach an arbitrary target. While the defenders of the target IOP concept might say that one could just raise the target IOP number or range in this circumstance, there is an alternate explanation of what they are actually doing. Most experienced practitioners make treatment decisions based upon a balance between the risks and benefits of a particular therapeutic step. So in the patient scenario described above, one would advance therapy only when the expected benefit outweighs risk for each step. In this particular case, the expected risk of trabeculectomy would be greater than the expected benefit and thus trabeculectomy would not be advocated. In glaucoma care, we generally start with less risky therapies such as medications and progress to those with greater risk such as laser and filtration surgery. There is also some evidence from early manifest glaucoma treatment study (EMGT)[24] and CIGTS^3^ that perhaps there are diminishing returns, on average, with progressive IOP lowering. Thus the marginal benefit from lowering IOP from 20 to 18 mmHg may be greater, on average, than the benefit of an equivalent 2 mmHg IOP lowering from 12 to 10 mmHg. This increasing risk and diminishing benefit is illustrated in Fig. 1. While the shape of these curves can be debated, one cannot argue against the approach. In all areas of medicine, treatment decisions are based upon the risk versus benefit of the next step. Glaucoma is no different. The target IOP concept, while convenient and useful in the rare circumstances when any and all risks will be tolerated to reach an IOP goal to preserve remaining vision, does not describe how most experienced practitioners initiate or advance therapy. Further, there have been no randomized controlled clinical trials that have shown that the use of a target IOP approach is advantageous to any other approach. Finally, setting a target IOP goal, when shared with the patient, can result in further exaggeration of the importance of IOP when the primary focus should be on visual preservation. The alternative approach of weighing risks and benefits is dynamic in that the expected risks and benefits of a particular therapy can change over time and treatment decisions should be made to reflect the present rather than the past disease state for an individual patient. This alternative approach actually reflects how most experienced glaucomatologists practice, regardless of whether or not they espouse the target IOP concept. In fact, decision making based upon weighing risks and benefits is how all physicians practice.

## Advancement of Therapy

One of the great mysteries in the medical treatment of glaucoma has been the lack of additivity of topical beta blockers to prostaglandin analogs when used separately or in combined preparations. So while topical beta blockers may be the second most desirable agents for initial therapy after the prostaglandin analogs, the beta blockers are not necessarily the best adjunctive agents to be used when additional therapy is needed in patients already receiving prostaglandins. The topical carbonic anhydrase inhibitors, in particular, have been shown to be beneficial adjuncts to prostaglandin analogs both during the day and night.[25,26] There may be significant interpatient variability in response to beta blockers, carbonic anhydrase inhibitors, and alpha adrenergic agonists as second agents following the use of prostaglandins. Because of this variability, it is generally best to use the two agents separately prior to using combined medications,[2] and monocular therapeutic trials may be more useful when adding second and third medications relative to the initiation of medical therapy with prostaglandin analogs.

In an era when laser trabeculoplasty is a perfectly reasonable first step for glaucoma therapy, the use of this procedure with argon and YAG selective laser trabeculoplasty (SLT) or diode approaches following the use of prostaglandin analogs or other first-line agents should not be overlooked. Despite the fact that laser trabeculoplasty is a procedure, the safety of this treatment places it within the realm of medical rather than surgical therapy. One of the greatest limitations of laser trabeculoplasty is the variability in the magnitude and duration of response. Patients should not be led to develop a false sense of security when having this procedure performed. It is not a cure and, in fact, is less likely to provide long-term IOP lowering than the use of prostaglandin analogs. In patients who do not keep regular follow-up appointments, long periods of time between visits may lead to significant periods of uncontrolled IOP if one relies on laser trabeculoplasty alone to treat glaucomatous disease. For all of these reasons, laser trabeculoplasty is likely a better adjunctive rather than initial therapy for glaucomatous disease.

## Maximal Medical Therapy

There was a time when maximal medical therapy included every available glaucoma medication including beta blockers, pilocarpine, and epinephrine. Over the past 20 years, there have been more classes of drugs introduced for glaucoma care than the prior 100 years. With multiple agents available in each class, there are thousands of potential combinations and the concept of maximal medical therapy has evolved.[27] Given diminishing returns with each additional medication, it is rarely beneficial for a patient to be receiving two or more glaucoma drugs at the same time. It is difficult to get an additional 2 mmHg IOP lowering when adding a second agent to a prostaglandin and the third agent likely adds less than the second. Nonmedical options should be considered in patients at moderate to high risk of vision loss from glaucoma who are already receiving two or three medications. Optimal medical therapy, which generally includes two or three medications, has replaced the concept of maximal medical therapy.[28]

## Follow-up

In general, the length of follow-up between glaucoma visits is determined by the severity of disease and the degree of control, the latter only being established with longitudinal follow-up.[23] Patients in whom a medication has been added or removed should routinely be seen within approximately 1 month of the change. Patients in whom satisfactory IOP control has been achieved should be followed at 3- to 6-month intervals with longer follow-up intervals, for those with less severe disease relative to those with more severe disease. Ocular hypertensive patients with normal optic nerves and visual fields should generally be followed at 6- to 12-month intervals. Those under treatment for ocular hypertension as well as those who have been followed without change for many years should be seen less frequently than those not receiving treatment and/or newly diagnosed patients, all other things being equal. Glaucoma patients should generally have careful optic nerve examination and visual field testing at least once a year, more frequently in cases with severe disease and/or rapid progression. It is generally helpful to obtain three or at least two baseline visual field tests at the time of initial diagnosis. As with IOP measurements, establishing a good baseline is critical for long-term follow-up. The same can be said for optic nerve imaging including photography where baseline measurements can be helpful in the years to come. Stereodisc photographs remain the gold standard for disease staging and for following structural optic nerve progression but advances in imaging have been dramatic and promise new standards in the future.[29,30]

## Concluding

There are several factors that predict glaucoma outcomes including stage of disease at the time of diagnosis as well as rate of progression. In recent years, much attention has been given to compliance, adherence, and persistence with glaucoma medications as predictors of the disease outcome. One factor that has been given remarkably little attention is follow-up. Lee *et al*. have shown that the there are many barriers to follow-up glaucoma care.[31] It is difficult to predict the course of a glaucoma patient but regular follow-up can allow the practitioner to adjust care depending upon individual disease risk, rate of progression, and response to therapy. Inadequate follow-up is thus a likely important prognostic indicator of the disease outcome. Patients who do worst, all other things being equal, are the ones who do not show up to see their physicians on a regular basis. In this regard, glaucoma is no different from any other medical disease.
